# Forest, Trees, Dynamics: Results from a Novel Wisconsin Card Sorting Test Variant Protocol for Studying Global-Local Attention and Complex Cognitive Processes

**DOI:** 10.3389/fpsyg.2016.00238

**Published:** 2016-02-23

**Authors:** Benjamin Cowley, Kristian Lukander

**Affiliations:** ^1^Brain Work Research Centre, Finnish Institute of Occupational HealthHelsinki, Finland; ^2^Cognitive Brain Research Unit, Institute of Behavioural Science, University of HelsinkiHelsinki, Finland

**Keywords:** attention, complex cognition, global-local processing, forced choice task, response time analysis, double filtering by frequency, object file theory, Wisconsin Card Sorting Test

## Abstract

**Background:** Recognition of objects and their context relies heavily on the integrated functioning of global and local visual processing. In a realistic setting such as work, this processing becomes a sustained activity, implying a consequent interaction with executive functions.

**Motivation:** There have been many studies of either global-local attention or executive functions; however it is relatively novel to combine these processes to study a more ecological form of attention. We aim to explore the phenomenon of global-local processing during a task requiring sustained attention and working memory.

**Methods:** We develop and test a novel protocol for global-local dissociation, with task structure including phases of divided (“rule search”) and selective (“rule found”) attention, based on the Wisconsin Card Sorting Task (WCST). We test it in a laboratory study with 25 participants, and report on behavior measures (physiological data was also gathered, but not reported here). We develop novel stimuli with more naturalistic levels of information and noise, based primarily on face photographs, with consequently more ecological validity.

**Results:** We report behavioral results indicating that sustained difficulty when participants test their hypotheses impacts matching-task performance, and diminishes the global precedence effect. Results also show a dissociation between subjectively experienced difficulty and objective dimension of performance, and establish the internal validity of the protocol.

**Contribution:** We contribute an advance in the state of the art for testing global-local attention processes in concert with complex cognition. With three results we establish a connection between global-local dissociation and aspects of complex cognition. Our protocol also improves ecological validity and opens options for testing additional interactions in future work.

## 1. Introduction

Hierarchies abound in models of cognitive processing. Processing of visual stimuli is hierarchical both at input, as scenes are composited from low-level visual features, and later at representation where directed attention can be global (requiring integration of visual features) or local (requiring focused attention). Object recognition and contextualization relies heavily on the integrated functioning of global and local processing (Hellige, 1993 p. 75), with lateral association to the cerebral hemispheres, and with a processing advantage for global stimuli (“global precedence effect”; Navon, 1977). As an isolated cognitive phenomenon, this has been well-studied. On the other hand for higher-level cognition such as knowledge work, global-local processing becomes a sustained activity implying a consequent interaction with executive functions. Such “ecological” interactions have not been much studied; indeed Logie et al. (2011) has referred to “a major lacuna in our understanding of complex cognition.” Thus, studying global-local attention alongside complex cognition brings up novel questions, of which we focus on two:

1. Are common global-local processing effects, such as global precedence, constant under changing conditions of adversity for subjective hypothesis formation, testing and updating?
2. Are there individual differences in preference and processing of various types of local/global stimuli, and do they affect processing speeds and learning response?

We are motivated by the increasing trend toward knowledge work through computer interfaces, which makes the processing of global vs. local levels of visual information in e.g., user interfaces, into a task-relevant internal state of the individual. It is thus an important construct to understand in both basic and applied terms. Most work in the area has focused on the isolated phenomenon of global-local processing, analysing behavior and imaging of parietal brain areas. While we have gained understanding of the mechanisms of global and local processing, most of the results use quite simplistic stimuli, and there has been little integration with other lines of work on cerebral hemispheric asymmetry or executive function. Thus, there is a need to expand the state of the art in two directions: to introduce more ecological validity, and to examine a wider picture of the cognitive functions (and associated brain networks) involved. For this work, and especially for application, novel approaches are needed to study the dissociation of global from local processing, and help move it out of the lab.

In this paper we present: a novel protocol to examine global-local dissociation in the context of executive functions (with source code plus stimuli); results from a laboratory experiment; a discussion of the outcomes and issues; and a look at future work. As mentioned, the overall aim includes examination of brain imaging data but this analysis will be reported in further work. Thus, within the scope of this paper, the analysis of behavioral results addresses two sets of questions: our primary research questions defined as hypotheses in Section 1.3, and a set of propositions to validate the protocol defined in Section 2.2.3.

### 1.1. Theoretical background

The question of how global differs from local processing has been investigated productively with theories proposed consecutively by Navon (1977); Sergent (1982) and Robertson and Ivry (2000). A consistent experimental observation has been that global processing induces relatively greater right hemisphere activation [measured by intra-cranial brain imaging and electroencephalography (EEG)], while the left hemisphere is similarly relatively more activated for local processing (Heinze et al., 1998; Fink et al., 1999; Han et al., 1999; Lux et al., 2004; Weissman and Woldorff, 2005).

Theoretical explanations for the bias tend to follow Robertson and Ivry's (2000) double filtering by frequency (DFF; but see Peterzell, 1998 for controversy regarding this theory's originality). In DFF each hemisphere enables relatively greater efficiency for processing low or high spatial frequencies, by first bi-laterally selecting an appropriate range of frequencies, and then at higher stages of perceptual processing, unilaterally performing low- or high-pass filtering. Thus, hemispheric bias is a function of efficiency and not capability. The DFF model accommodates several experimental observations:

- The response time to recognize global stimuli is less than for local stimuli.
- The task acts as a primer, such that hemispheric asymmetry is larger when the level to be attended is known (Heinze et al., 1998; selective attention), as opposed to when the level must be found on each trial (divided attention).
- The hemisphere bias for hierarchical level is co-associative with relative spatial frequency (Sergent, 1982).
- The *scale* of global vs. local features is relative, so that a stimulus with spatial frequency of e.g., three cycles per phase is considered “local” when compared with a one cycle stimulus, and becomes “global” when compared with a nine cycle stimulus (Fink et al., 1999).

As a theoretical consideration, DFF theory is complemented by object-file theory (Kahneman et al., 1992), and this relationship has been examined recently by Valdés-Sosa et al. (2014). The latter work suggests that frequency filtering is responsible for the extraction of object features, whereas the object-files are responsible for retaining the object-file identity and parameters, and to an extent, for guiding the top-down selection of spatial filtering frequency. Valdés-Sosa et al. (2014) suggest that a single object-file cannot hold information from different hierarchical levels. They claim that attending to local features segregates the whole into its constituent parts, destroying the object-file for the global form; integrating local features into a global form does the opposite, abandoning the object-files for the local features (Valdés-Sosa et al., 2014). Only a limited number of object-files can be handled by attention at a single time (Wheeler and Treisman, 2002; Saiki and Miyatsuji, 2009), i.e., the storage of object-files is resource-limited. Thus, object-file theory points directly at the possible interaction executive functions may have with global local processing, as maintenance of a particular object file in the face of competing demands for attention requires active executive control.

Many tasks from the global-local literature could be said to involve executive functions, but the associated research questions were not generally interested in high-level cognition. Literature using global-local matching tasks to study executive functions includes, for example, a study of executive advantage in *N* = 151 bilingual children (Bialystok, 2010), using a version of the task from Andres and Fernandes (2006), whose own study examines dual-task interference with global precedence. Both studies show some interaction effects, but are restricted by population and cognitive scope, respectively. We attempt to incrementally broaden the cognitive scope of study, by adapting the Wisconsin Card Sorting Task (WCST).

WCST is a well-studied broad test of executive functions, as it was designed to assess cortical prefrontal function, requiring a smooth combination of active cognitive processes such as working memory, rule deduction and updating, (non-binary) decision making, and visual processing (Cinan and Tanör, 2002; Nyhus and Barceló, 2009). WCST acts as a “card game,” where the subject has to match target cards to reference cards based on a periodically changing matching rule that the user has to discover through trial-and-error feedback. Once found, a matching rule must be maintained for a number of repetitions until the next rule change. According to a literature review in Cinan and Tanör (2002), WCST evokes the following executive functions: maintenance of disparate information including current and recent feedback plus predictive hypotheses; regulation and reorganization of responses to environmental cues; concept formation; and inappropriate response inhibition. Although WCST has been criticized in its role as a neuropsychiatric test of prefrontal function, such a test is not the purpose of this study, and we use and adapt only the task structure of WCST. The relevant characteristics of the task structure are: the trial-and-error based search for the correct matching rule; non-binary choice task; and maintenance of the discovered rule across a block of changing-stimulus trials.

### 1.2. Global-local dissociation protocol

Our primary aim is to explore and dissociate global from local processing in the context of executive functioning. To do this we developed a novel protocol, built on established methods. The task elicits non-instructed global and local attention, across blocks of trials whose overall structure elicits a set of executive functions similar to WCST. We thus present the Wisconsin-ish Global-Local Dissociation (WishGLD) protocol, inspired by WCST.

Given the broad support for the spatial frequency theory, we designed WishGLD to hold this visual feature constant, i.e., minimize spatial frequency difference between conditions for each level. The protocol was then designed to specifically address three important constraints: first, manipulate selective vs. divided attention; second, increase the complexity of stimuli to produce a *more* ecologically valid information processing task; third, control for known confounds.

#### 1.2.1. Selective vs. divided attention

This protocol is the first (to our knowledge) to use multiple stimuli per hierarchical level during each trial. Combined with the WCST task structure, multiple stimuli per level allows WishGLD to encapsulate both divided and selective attention. Participants must use trial and error to discover which matching rule is in effect, i.e., which level to look for target stimuli, inducing level-switching focus (divided attention). When the rule is found it remains fixed for a block of trials, inducing consistent focus (selective attention). With four matching rules and these relatively long “rule search” and “rule found” sequences, WishGLD induces the following non-trivial requirements for executive functions (in order of occurrence): concept formation; maintenance of disparate information including current and recent feedback plus predictive hypotheses; regulation and reorganization of responses to environmental cues; and inappropriate response inhibition.

#### 1.2.2. Ecological validity

**Figure 1:**
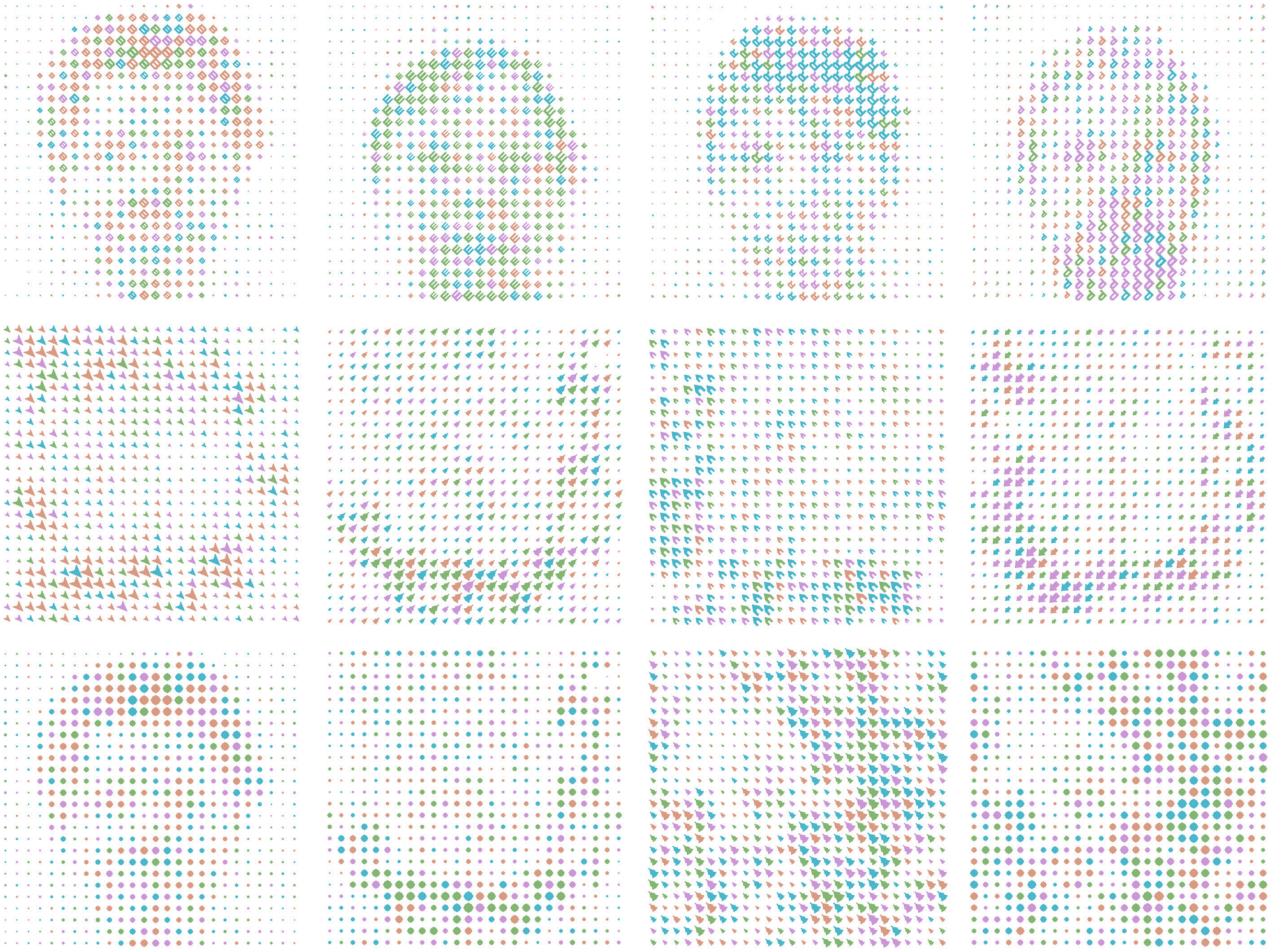
**Example stimulus cards, illustrating all the various stimulus classes in a subset of the conditions**. **Top row**: global faces built of letters. **Central row**: global letters built of shapes. Columns in top two rows agree in dominant (global) color and (local) orientation. **Bottom row**: reduced-stimulus conditions. From left: patch replaces local stimuli for global face, global letter; noise with no dominant color replaces global stimuli for local shape, local patch. The bottom right card contains only (global) noise and (local) patch, and was used only in a baseline condition.

The ecological validity of the task is improved over previous work by three means. First, the task structure induces global or local processing by participant's choice of strategy and attended stimulus content, as opposed to induction by instruction. Second, the choice and design of stimuli (shown in Figure 1):

- Our stimuli were designed with intrinsic noise, which helps to simulate real-world conditions and balance discriminability between and within stimulus classes.
- Faces were our first stimulus choice because the evolved functionality for face processing in humans creates the strong likelihood for integration of the local stimuli to a global percept even under noisy stimulus conditions (Kanwisher and Yovel, 2006).
- Color was chosen as another global stimulus because color is a fundamental feature of modern user interfaces, and as colors can readily be made equiluminant and equally perceivable.

Third, the use of two stimulus characteristics per level, with more naturalistic stimuli (e.g., faces and colors) creates a rich information space closer to modern user interfaces. We chose global and local letters to represent the additional characteristic, as explained in the next section.

The last stimulus choice was orientation of local shapes/letters. Orientation was chosen because it enables the creation of four readily-distinguished features for the discrimination task in WishGLD. Using four orthogonal orientations helps to reduce intra-class noise for the matching task without affecting stimulus noise.

#### 1.2.3. Confound control

The so-called “semantic confound” for global-local processing is due to the fact that the left hemisphere is known to dominate in semantic processing (see Binder et al., 2009), which can bias results when stimuli have semantic value. This confound has previously been studied by, for example, Kéïta et al. (2014). Given the novel nature of our task, we wished to address this confound within the current study. Thus, WishGLD stimulus conditions include both letters and faces at global level, and letters and shapes at local level. When designing for confound control, variables associated with spatial location, motivation, or emotional effects were held fixed to maintain a tractable number of conditions (some non-comprehensive testing was still conducted for spatial location). The WishGLD protocol also supports the option to be easily altered to manipulate these effects (see Section 4.4).

### 1.3. Research questions

Our hypotheses focus around the two “novel questions” raised above.

H1a,b,c test variables acting as a proxy for difficulty of participant's strategy formation, testing and updating. First, H1a, when individuals need to formulate a hypothesis under conditions of uncertainty, they may follow a test strategy that seeks more confirmatory evidence (a proactive form of hypothesis unpacking; Tversky and Koehler, 1994). Second, H1b, it is known that global precedence disappears when global information is presented with a delay >80 ms relative to local information (May et al., 1995); we propose that global precedence also diminishes if the global matching task is more difficult, inducing a “natural” delay. Third, H1c, because there is a cognitive cost for maintaining in working memory the elements of a strategy across a block of trials (e.g., the current and alternative rules), we propose an advantage when switching to a rule at the same level. Formally, we have:

- H1a - more errors made during search will predict worse “rule-found” performance, in terms of response time metrics.
- H1b - the global precedence effect (on response time) will decrease proportionally to increasing search errors.
- H1c - switching between rules on different levels will incur a performance cost, such that (local-local/global-global) changes have lower costs than (local-global/global-local) changes.

H2 tests variables related to performance subjectivity. Studies have shown that variability in different patient groups (autism, schizotypy, left hemisphere damage) is linked to heightened perceptual sensitivity to either local or global features (Robertson and Lamb, 1991; Plaisted et al., 1999; Goodarzi et al., 2000; Mevorach et al., 2006). Based on observation and reports from our pilot studies, we expect that perceptual variability also manifests in the healthy population, therefore we have:

- H2 - inter-individual variability in response time performance between stimulus classes will correlate with subjective difficulty ratings of the different target stimulus classes.

In the Section 3 we describe the details of the laboratory experiment, Section 2.1, and protocol design, Section 2.2. In Sections 3 and 4, we describe the outcome of the experiment and implications for the state of the art, plus intended future work. As it is a novel protocol, we also describe the stimulus preparation and validation procedures in Sections 2.2.2, and 2.2.3. Readers who are uninterested in the protocol *per se*, may choose to skip these sections, and also Section 3.2 Validation Results.

## 2. Materials and methods

Here we give a detailed account of design and implementation of the experiment and of the WishGLD protocol. We first designed and pilot tested WishGLD; we then conducted a controlled laboratory experiment, where participants' physiology was recorded while performing the protocol. Analysis reported herein focused on behavioral and validation questions.

### 2.1. Experiment design

#### 2.1.1. Participants

The recruitment process involved advertisement by mailing list in the Helsinki area. Recruitment inclusion criteria included good state of health; age range between 18 and 60; strong right-handedness (to provide uniform brain imaging results); mother tongue Finnish (for instruction purposes); vision and color-vision normal or corrected to normal. Exclusion criteria included hairstyles, scalp conditions or implants which would be obstructive to the physiology recording; use of medication with neurological effect, such as anti-depressants; and prior psycho-pathological diagnoses.

Twenty-five participants (19 female; age *M* = 28, *SD* = 8.5; average education level: university degree) were included in the final study, and rewarded with non-remunerable vouchers. They were emailed instructions and the Edinburgh Handedness questionnaire (*M* = 92, *SD* = 15).

The protocol followed the Declaration of Helsinki for the rights of participants and study procedures. An ethical approval of the present research protocol for all participants was obtained from The Ethical Committee of the Hospital District of Helsinki and Uusimaa. All participants were briefed on their rights, and signed informed consent.

#### 2.1.2. Recording

Participants were brought to the lab in the morning between 07:30 and 11:45. They were first briefed on the task, and asked to complete the Karolinska Sleepiness Scale (KSS; Akerstedt and Gillberg, 1990; *M* = 3.7, *SD* = 1.5) and the relevant part of Ishihara test for color blindness (Clark, 1924) (no failures).

As mentioned, one aim of the study was to investigate the relationship between psychophysiology and global-local processing; however this data is not reported herein. Thus, we give only a brief description of the recording set-up used without detailed notes on apparatus. Participants were dressed in a 32-channel electroencephalography cap, four electrodes about the eyes for electro-oculography, two electrodes on the torso for electrocardiography, and two types of electrodermal activity sensors on the non-dominant hand (Torniainen et al., 2015).

After electrode dressing participants were seated on a comfortable chair with head rest, in a sound-proof isolated room, 1 m from a 1920 × 1080 Samsung SyncMaster P2770FH screen occluding 37.8° of visual angle. Stimulus cards presented on the screen occluded 9.1°; each “pixel” on a card occluded ≤ 0.46°. Both hands sat on arm rests and a response pad was set at their right hand.

Two baseline recordings were made: one for a wildlife video, one for response-free presentations of the WishGLD trial with noise × patch cards, i.e., cards with no information at global or local level of stimulus. The latter baseline had four trials, 15 s∕trial. Participants then performed five practice sets; before and during practice they were given precise instructions on the task, including the strategy suggestions. After practice, the experimenter withdrew from the room and gave the permission to begin testing. Test durations were around 1 h (*M* = 62 min, *SD* = 9.7 min).

**Figure 2:**
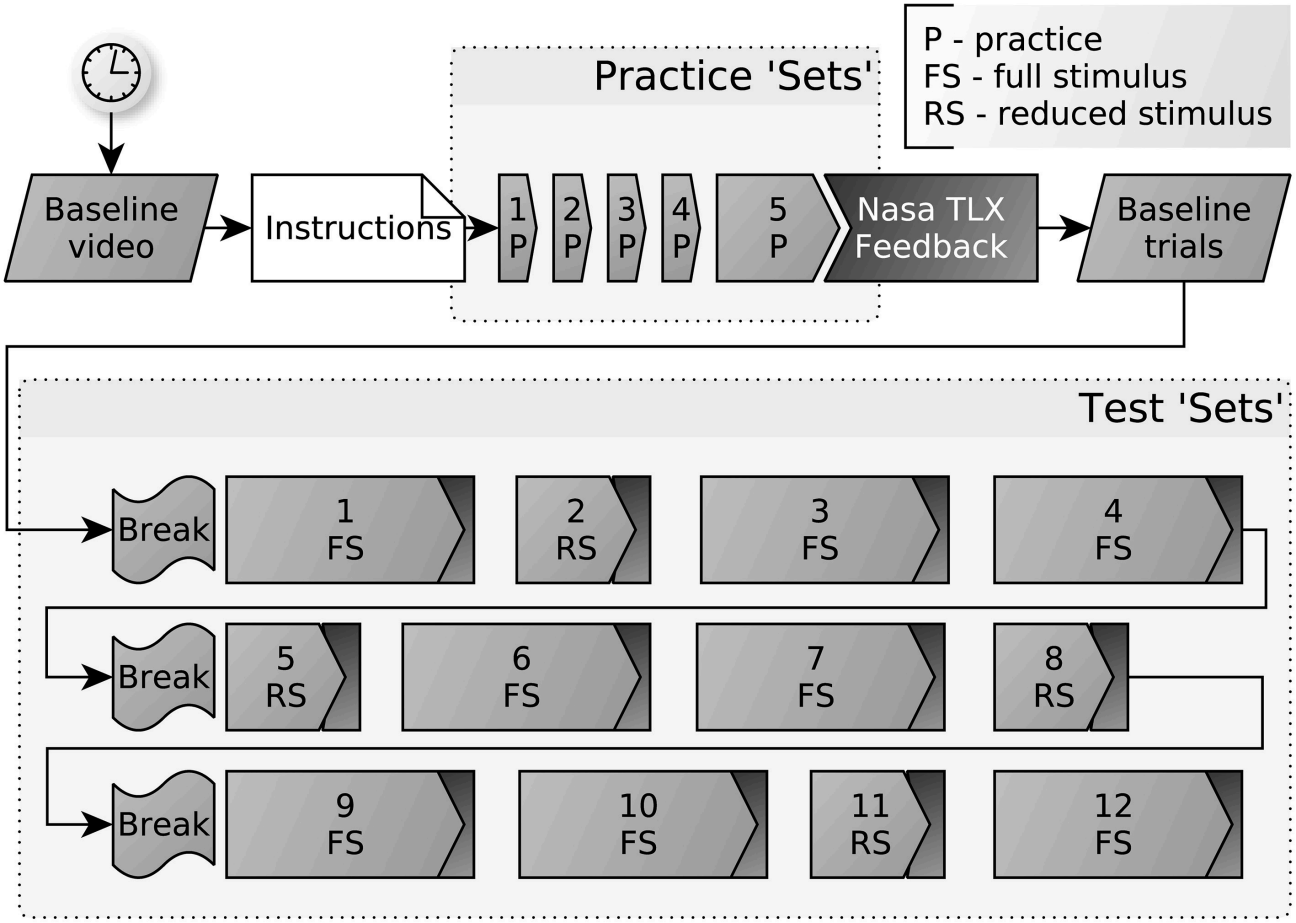
**Structure of the protocol, with five practice sets and 12 test sets**. Each set consists of a number of blocks. Each block has a constant matching rule, over a number of trials. Every test set is followed by an abbreviated version of the Nasa Task Load Index (TLX): effort and frustration items.

The complete protocol structure is described below (in Section 2.2.1), and illustrated in Figure 2. During testing, one third of sets used “reduced-stimulus” cards, with either local or global information missing, which were designed to provide controls for the brain imaging analysis, much as the second baseline. Again, this analysis is not presently discussed and so for the purposes of behavioral results, these sets can simply be considered as interstitial “lower difficulty” tests. The data from these test sets are excluded from the datasets analyzed herein.

### 2.2. Protocol design

**Figure 3:**
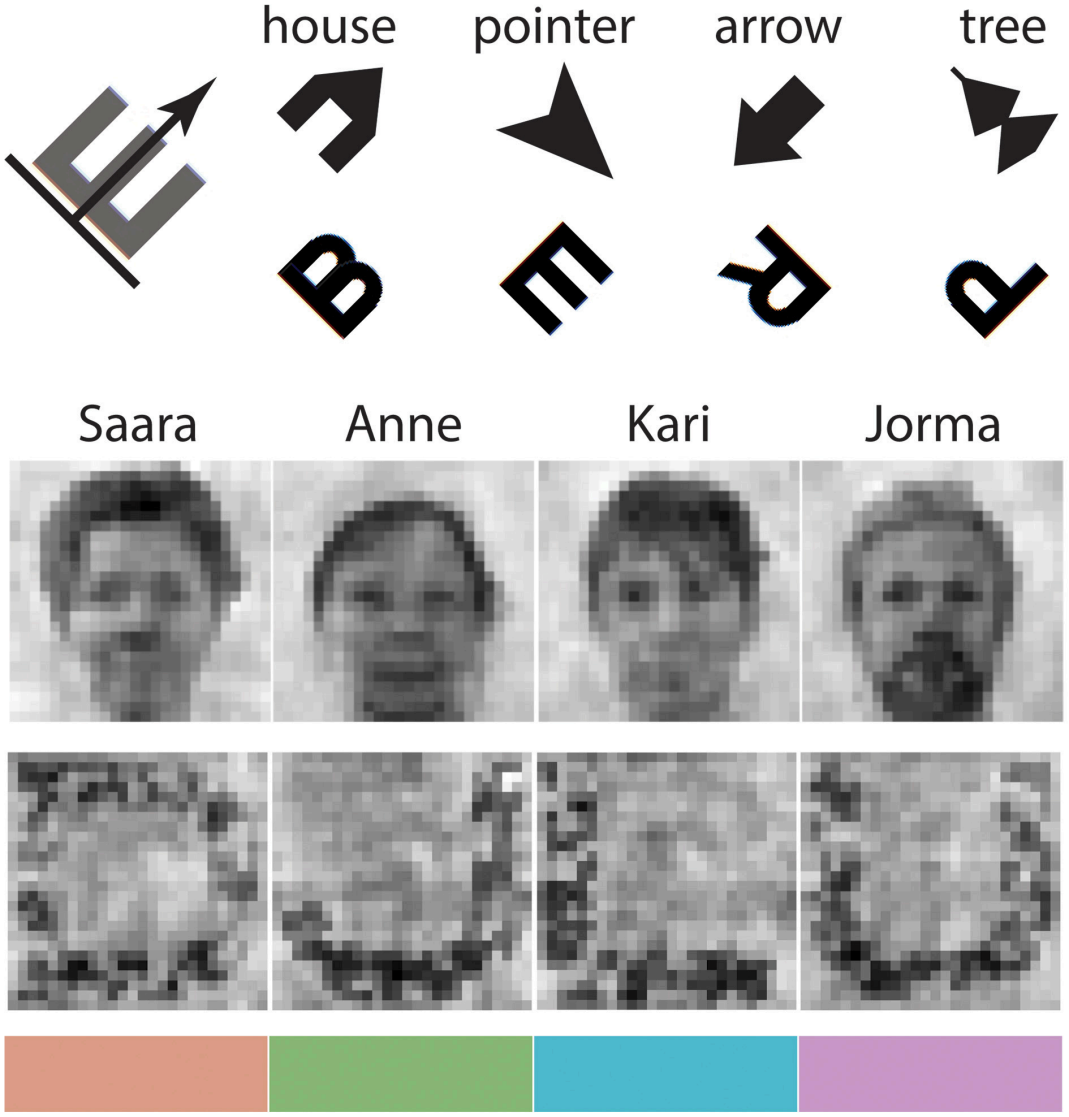
**Basis stimuli used for processing (excluding noise and patch), also shown as example images in participants' instructions**. **Top**: oriented local letters and shapes. Names were given to each shape as a “strategy” to help balance the difficulty of remembering against that for letters. **Top left**: a “strategy” for mentally orienting the letters was suggested; it was explained that this example should be applied to all four letters. **Center**: participants were shown down-sampled portraits and global letters to illustrate the basis of their stimuli. Portraits were named, again as a “strategy” to help remembering. **Bottom**: four perceptually equal colors.

The WishGLD protocol is a *variant* of WCST: there is a novel set of “cards,” each with two local and two global visual features, and a simple response paradigm for minimizing required motor actions. The subject performs the task at his own pace, while instructed to operate as quickly and effectively as possible. Every block of trials has an undeclared “matching rule” (*L1.obj, L2.ori, G1.obj, G2.col*, see Table 1), so that the participant must match the target card feature to the feature from one of four reference cards. Features correspond to some stimulus property at a local or global level, see Table 1. The participant must always deduce the current rule on the basis of feedback (right/wrong) given after the selection has been made. During practice she must learn the requirements for remembering the current rule, noting when the rule has changed, and following some trial-and-error strategy to find the new rule. Figure 3 shows visuals used in the instructions to clarify the task and suggested strategies.

**Table 1:** **Global (G) and Local (L) matching-rule names and associated hierarchical features and rationales**.

**Rule**	**Feature**	**Rationale**
L1.obj	“Pixel”: shape or letter	Each “pixel” in the image is from a set of four shapes or letters, visually balanced so that local focus is required to differentiate them
L2.ori	“Pixel” orientation	All “pixels” share a dominant orientation (NE, SE, SW, NW), which should be indistinguishable without local focus
G1.obj	Image: face or letter	“Pixel” sizes vary to create a halftone representation of a face or letter, the participant must integrate the whole to identify the feature
G2.col	Dominant color	Each “pixel” is colored from a palette of four equiluminant colors, of which one is most prevalent: integration is required to assess this

#### 2.2.1. Protocol structure

The test protocol has a forced-choice task structure, with a required “correct response” count of 720 trials plus a non-fixed number of incorrect trials. The total of trials is divided into 12 sets, of which eight were “full” sets and four were “reduced” sets. This scheme allows various control comparisons to be made; for example estimates of cortical hemispheric asymmetry can be made for when the participant has only one level to attend. Details of “reduced” sets are presented for completeness. Each set corresponds to one of the eight separate stimulus conditions presented in Table 2.

**Table 2:** **Test conditions**.

**Stimulus-category**	**Condition**	**Set repetitions**	**Global 1**	**Global 2**	**Local 1**	**Local 2**
Full	1	2	Faces	Colors	Shapes	Orientation
	2	2	Faces	Colors	Letters	Orientation
	3	2	Letters	Colors	Shapes	Orientation
	4	2	Letters	Colors	Letters	Orientation
Reduced	5	1	(Color-balanced) noise		Shapes	Orientation
	6	1	(Color-balanced) noise		Letters	Orientation
	7	1	Faces	Colors	Local (circular) Patches	
	8	1	Letters	Colors	Local (circular) Patches	

*No.s 1–4 are “full-stimulus” conditions, each of which was presented in two separate sets (to allow breaks and counter-balancing); no.s 5–8 are “reduced-stimulus” conditions, which have stimuli only on local OR global level, and were presented in one set each*.

Every set is sub-divided into blocks. In each block all trials have the same target rule. When a block changes a new rule is randomly chosen, never the same rule twice. Each block has a randomly permuted correct trial count from five to seven, i.e., the number of trials which must be answered correctly before the next block is triggered and the rule is changed. Trial-count and rule per block are controlled by pre-generated configuration files, one for each set (but see Section 4.4). In full-stimulus sets there are 12 blocks and 72 correct trials; reduced-stimulus sets have six blocks. There is no limit to the number of incorrect trials, nor is there any requirement for correct responses to be consecutive. Full-stimulus sets therefore have a “search space size” (SSS) of three, because three rules could potentially replace the last rule; reduced-stimulus sets have SSS = 1 (the exception is always for the first block of a set, where full SSS = 4 and reduced SSS = 2).

Order of presentation for the full-stimulus sets is counter-balanced by application of an 8 × 8 latin square for the “full” sets. Reduced-stimulus sets are counter-balanced with a 4 × 4 Latin square, and presented after full-stimulus sets 1, 3, 5, and 7. The complete counter-balance index thus has 12 rows and eight columns, and is repeated after every eight participants, implying a repetition count of *N*∕8.

In addition, there are five practice sets: four reduced-stimulus sets with two blocks each, and one full-stimulus set with four blocks. This adds another 71+ trials.

**Figure 4:**
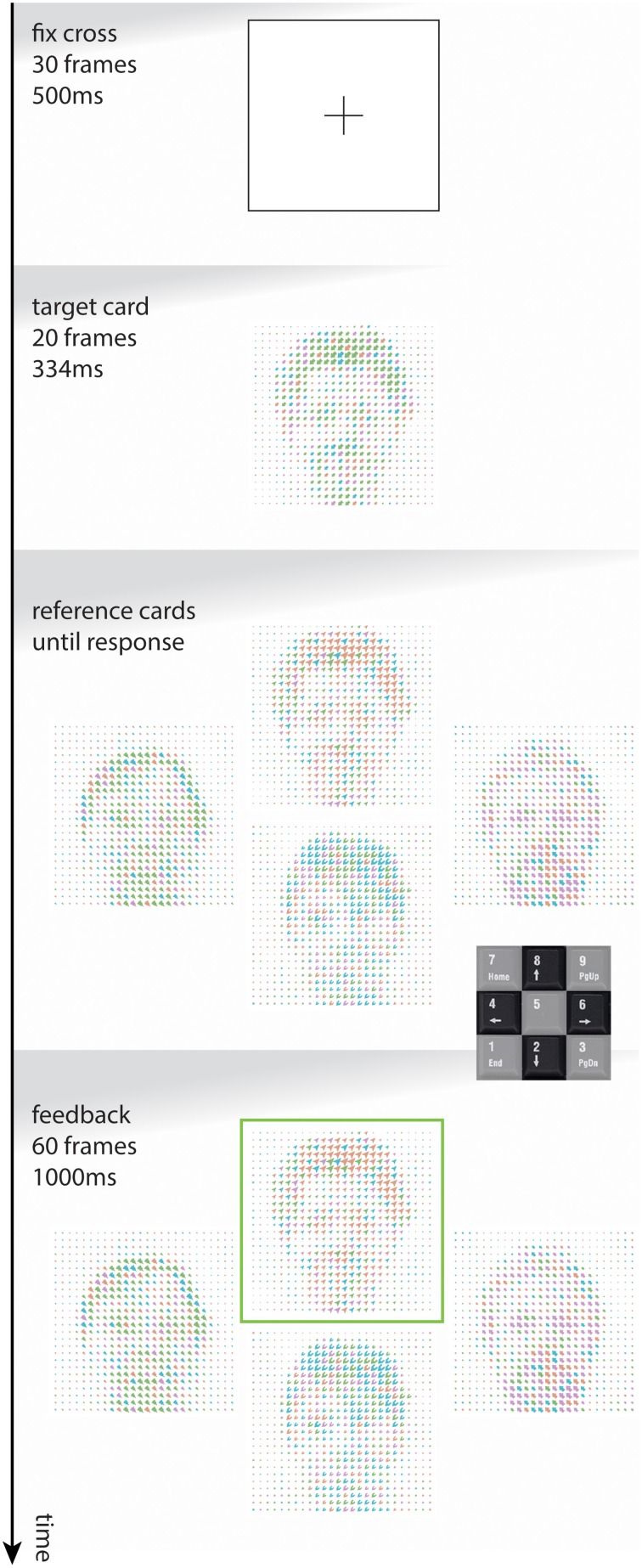
**A schematic of a single trial**. Numpad shown for illustrative purposes. Matching rule for the task shown is G1.obj (face).

Each trial has the temporal format: fixation cross (30 frames, 500 ms), target card (20 frames, ~333 ms), reference cards (response driven duration), feedback (60 frames, 1000 ms). The format is illustrated in Figure 4. Responses are made by “arrow” keys of a numpad. For the experimental data collected, response times for all test trials ranged from 0.05 to 23.8 s (*M* = 1.2, *SD* = 0.9).

Target presentation time is long enough (>300 ms, Kutas et al., 1977) to allow any *one* visual feature to be checked and consciously processed, but not longer to prevent switching of focus between levels. Between-level attentional blink studies, e.g., Dale and Arnell (2010), suggest that detecting features on the opposite level would require >400 ms presentation times. While presenting the target separate from the reference cards is different to the classic WCST, we need to control the subjects' attention at the time of presentation to enable clear inferences about visual processing. While limiting the presentation time of the stimuli, we opted for a free response window to avoid introducing a false ceiling effect.

For each of the first four stimulus conditions listed above, there are 256 cards as the combination of 4^4^ stimuli, two global and two local. For each test set, four reference cards are selected randomly from 256, with the constraint that all four cards must have disjoint stimulus combinations. Due to this constraint, any given target card will match each of the reference cards for a separate stimulus, or in other words, the target will match only one of the reference cards for the current rule (e.g., in Figure 4 the face is matched). For each trial, a target card is selected randomly, and reference cards are displayed in an arrangement which remains fixed throughout a set.

**Figure 5:**
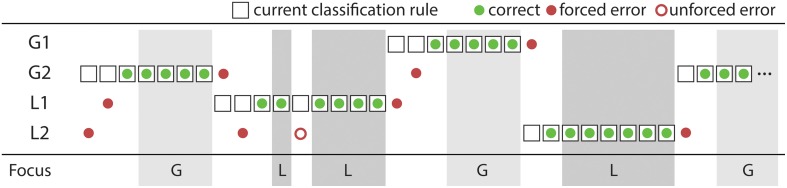
**A schematic of a subset of blocks**. Errors directly after a rule change are forced; errors within a “rule found” sequence are unforced.

A portion of a full-stimulus set, containing four full blocks, is illustrated schematically in Figure 5. The current rule is shown by sequential boxes; correct trials are green, incorrect are red; and the subjective global-local focus is illustrated at the base.

After every set, the participant is asked two questions from the Nasa Task Load Index (TLX), rating their subjective effort and frustration for that set. After the test, the participant rates the subjective difficulty of each stimulus from hardest (one) to easiest (six): first by nominating the easiest and hardest within local and global categories, and then ranking all six stimuli together.

The protocol is implemented in the Psychopy psychophysics environment v1.81.00 for Python Peirce (2007), and precise timing is recorded by log file entries generated for each of fixation, target, reference, response and feedback. Psychopy was also used to generate the stimulus cards as described next.

#### 2.2.2. Stimulus preparation

Our stimulus creation method follows Knowlton and Harmon (1972). An original image, such as a photograph of a face, is down-sampled to 26 × 26 pixels, giving an array of 676 grayscale values (as in Figure 3). In a 512 × 512 blank white “card,” a single local level stimulus (e.g., shape, letter) is mapped to each of these 676 values on a 26 × 26 grid; grid squares have ~20 pixels per side. Stimulus size is in inverse proportion to the pixel gray value, mapping gray levels to 20 preset sizes of stimuli; size scales linearly. Thus, for the local shapes, whiter areas of the global image have smaller shapes and relatively little coverage, and dark areas have larger shapes and more coverage. The result is a Knowlton-style rastered global image, built of identical copies of a local image of varying size. We will refer to the process as “additive rasterisation” (AddRas), because the technique adds information after down-sampling.

There are six “structural” classes of stimuli used as targets within the task: global faces, letters, and noise; local shapes, letters, and patches. Two more classes are “non-structural”: global (dominant) color, and local orientation. Each class is designed to meet the following criteria, which were chosen to facilitate a valid test, while also preventing discrimination confounds. The first two, most basic, criteria are related to hierarchical levels.

1. Local stimuli should be distributed across the card, such that they: contain high spatial frequency information; require focused attention for recognition; do not betray information about the global stimulus.
2. Global stimuli should have mainly low frequency information and require integration of the whole stimulus for recognition.
3. Visual feature differences between cards must be minimized, so that, e.g., the luminance and spatial frequency spectra of every card is the same, whichever local or global classes it contains.
4. Within-class discriminability of stimuli must be equalized *between* classes, e.g., have local shapes which are about as easy to discriminate from each other as local letters.
5. Point 4 must be facilitated also for the non-structural stimulus classes, e.g., that orientation of letters should be no harder to perceive than orientation of shapes.
6. Between-class discriminability must be equalized for global letter vs. face and local letter vs. shape stimuli (to allow fair comparison for the question of semantic value in global-local processing)^1^.

When processing original stimuli, in order to meet these criteria and balance the cards, metrics of the visual feature space were calculated using tools from the SHINE toolbox (Willenbockel et al., 2010) in Matlab 2012b v8.0, including the Structural SIMilarity (SSIM) metric (Wang et al., 2004). SSIM is used because it is designed to estimate image similarity from structural information, inspired by the human visual system. It thus tends to create intra-class estimates of image (dis)similarity which give perceptually valid results. In addition to using such objective metrics, performance data and subjective ratings were obtained from five pilot testers who performed the protocol to test candidate versions of stimuli.

Four *global face* stimuli were derived from a set of 19 grayscale portrait photographs (8f, 11 m) of typical Finnish office workers. Portraits were taken under controlled lighting conditions, and scaled to co-align the pupils. Three outliers greater than *M*± 2 SD in luminance and spatial frequency were rejected. The four card test set was selected by clustering the remaining 16 stimuli within their mutual inverse SSIM “space,” which is the co(inverse) SSIM matrix. We took the tightest cluster, which held the four maximally distinguishable faces; this was done because the faces tend to lose some ease of discrimination after AddRas. The four faces were passed through the SHINE matching process, with parameters set to “whole image” (include background), histogram and then spectrum matching, and SSIM optimization (for details see Willenbockel et al., 2010). A blank white card was included in this SHINE process, to create a “reference card” for the visual feature space of the portraits, to be used in matching other global stimuli (see below). The four portraits were then identical in visual feature space, and the AddRas process was applied to create global face cards composed of local shapes, letters, and patches.

*Global noise* cards were created by compositing the reference card with binary noise at several values of cycles-per-width from 8 to 64, to inject additional high spatial frequencies, and balance the spectra with other global stimuli.

All letters are based on the Sloan font (Pelli et al., 2009), because it is well-documented in the psychophysical literature and designed to be legible.

Four *global letter* cards were derived from the complete Latin alphabet by clustering in the mutual SSIM space. This was the opposite strategy to the natural face stimuli, because the letters contain significantly more high frequency power, which tends to be well-preserved in the AddRas process and enhances discriminability. A four letter set—D, J, L, U—was selected due to its visual feature profile and because the rounded shapes (in Sloan font) match well to the low frequency data of the face stimuli. The four selected letters were rendered within a 448 × 448 pixel boundary, to match the average spatial extent of faces, and padded with whitespace to 512 × 512. Letters were composited with binary noise at 5 cycles-per-width to break up their distinctive outline and reduce high frequency power, congruent with the criteria for global stimuli. As with the noise card, letter cards were composited with the reference card to balance luminance and low frequency power against the face stimuli. SHINE was not used for letters because it tended to propagate high frequencies from the letter's own outline across the image; after the reference composition, it was not necessary anyway.

To create the non-structural stimulus of *global color*, local stimuli in a card were colored with a palette of four equally divergent shades from the Hue-Chroma-Luminance (HCL) color space, which is perceptually based. We derived these following Zeileis et al. (2009), using their package **colorspace** for the R environment for statistical computing, v3.1.4 R Core Team (2013). Color was assigned to each local stimulus according to a probabilistic algorithm, which ensured that 50% of the total ink on the card was assigned to a dominant color, and the rest was divided evenly among the other three colors. The chosen colors translate to hexadecimal as below; they are shown in Figure 3, and as applied in Figure 1:

- color 1 = #DB9D85
- color 2 = #86B875
- color 3 = #4CB9CC
- color 4 = #CD99D8

Four *local shapes* were drawn directly in Psychopy using ShapeStim objects. They were designed to have identical luminance, contrast and spatial frequency profiles, while being mutually discriminable and facilitating the non-structural stimulus of orientation: thus arrow-like shapes were preferred. Simple initial shapes were perturbed by extending corners and adding concavities, increasing high spatial frequencies to give them a similar spectral profile to the letters.

Four *local letters* were selected from a reduced Sloan Latin alphabet, where nine letters were first rejected based on internal or shared symmetry, which would interfere with recognition of orientation. Selection was again by clustering in SSIM space. The chosen four letters—B, E, R, P—were thus very similar in luminance which meant the AddRas process could produce equiluminant cards with letters of similar font size. In addition, the four letters produce a co-SSIM matrix closely resembling that of the local shapes: in other words, the four stimuli in each class have intraclass visual similarity matrices that are approximately equal. This is important to make discrimination between separate stimuli about as easy for local letters as for local shapes (at least in terms of their visual features).

The non-structural stimulus of *local orientation* was achieved by rotating local stimuli by 45, 135, 225, or 315° (NE, SE, SW, NW). We chose not to alter the letter stimuli with a pointer, in order to preserve their canonical shape for recognition (but see Section 4.4).

*Local patch* cards were created by using a circular patch in the AddRas process instead of an oriented shape or letter, again designed to match luminance of the whole card.

After creation of global and local features and application of AddRas, the percentage of ink on each card was 17 ± 0.8%.

Finally, to improve comparability of stimulus-class processing, we suggested particular stimulus-recognition strategies to participants, in their written and vocal instructions. We assigned names to faces and shapes, because letters are already known by name and there is a memory component in the protocol since the target card disappears before the reference cards appear. To compare two oriented local letters, a common strategy of pilot testers was to imagine the letters drawn horizontally in the normal way, and then mentally rotate them in place. This is quite inefficient compared to the naturally oriented arrow-like shapes, so a strategy was suggested that participants imagine an arrow pointing toward the front of the letter, indicating the direction (see Figure 3).

Figure 1 shows example stimulus cards including all four global faces and letters, all four colors, all four local shapes and letters at orientation, noise and patch-type cards.

#### 2.2.3. Protocol validation propositions

Validation addresses the burden of proof of operation. We must first ask: when the task is to match the value of a stimulus at global or local level, is task performance independent of the target stimulus category? And complementary: is task performance independent of the stimuli which co-occur with a target stimulus? Ideally both of these questions should be answered positively, so that choice of stimulus does not bias task performance. Wherever the answers are negative, the effect of the stimulus condition will combine with the task condition, giving a potential confound but also potentially interesting results.

We make the following propositions to address these questions by testing against our experimental data. Naturally, when testing the expectation that two datasets are equal, we can never find confirmatory evidence that the difference between them is zero. We only expect to find lack of evidence to support their difference. In absolute terms the stimulus classes are all different, but it is enough that the difference is not significantly large.

- Pr1a - performance during rule G1.obj integration-task blocks (global processing) will be unaffected by target stimulus condition, comparing faces with letters.
- Pr1b - performance during rule L1.obj focal attention blocks (local processing) will be unaffected by target stimulus condition, comparing shapes with letters.

For the complementary question, the rules L2.ori, G2.col have targets—orientation and color—which co-occur with two separate types of stimuli *at the same level*. Within this complementary question, there are also potential inter-level effects: can stimuli at another hierarchical level act as distractors when not attended? We have worked to balance the visual properties and strategies of processing between such stimuli. Thus, non-target stimuli should not affect performance, and we predict:

- Pr2a - performance during G2.col color-target blocks will be unaffected by same-level stimulus condition, comparing faces with global letters.
- Pr2b - performance during L2.ori orientation-target blocks will be unaffected by same-level stimulus condition, comparing shapes with local letters.
- Pr2c - performance during G2.col color-target blocks will be unaffected by other-level stimulus condition, comparing shapes with local letters.
- Pr2d - performance during L2.ori orientation-target blocks will be unaffected by other-level stimulus condition, comparing faces with global letters.

G1.obj and L1.obj rule blocks also have variation of other-level stimuli; however given that the *N* of each of these rules is halved by the presence of two stimulus conditions, we consider that Pr2c,d already have more statistical power than could be obtained from the G1.obj or L1.obj data.

We also examine between-rule effects, based on three observations. First, global processing is often cited as faster than local. Second, it is well-known that color processing occurs relatively early in the visual system. Third, orientation of our (relatively complex) shapes and letters clearly is ontologically more complicated than their recognition. As we have introduced no specific manipulation to contradict these effects, we predict:

- Pr3a - rules G1.obj, G2.col induce faster response times than rules L1.obj, L2.ori.
- Pr3b - rule G2.col will induce faster response times than G1.obj.
- Pr3c - rule L2.ori will induce comparatively worse response times than L1.obj.

As spatial location has been implicated as a confound in earlier work (Lux et al., 2004), we wish to test whether there is an effect of lateral presentation of the reference card in WishGLD. There should be no effect because all reference cards are presented as an integrated stimulus and not to visual hemifields.

- Pr4 - performance is unaffected when the target-matching card is presented in the right or left position among the four reference cards, compared to top or bottom.

It should be expected that after repeated exposure to the stimuli, participants will experience a learning effect on their performance, at least with respect to speed of response.

- Pr5 - for both attention levels, we expect to see reduction in RTs due to learning in both short-term, over a single block, and long-term over the whole test.

### 2.3. Statistical analysis

Analysis was performed using the statistical computing environment R v3.1.4. For all hypotheses and validation propositions, the term “performance” refers to the block-wise Dependent Variables (DVs): response time (RT), response time variability (RTV), and accuracy *d*. For RT and RTV performance, lower is better; for accuracy, higher is better. Hypotheses or propositions not defined for “performance” are defined for a specific DV. RT and *d* are relatively straightforward measures of performance, justified and interpreted as the reliability of responding in terms of speed and freedom from error. It has been suggested that RTV is inversely related to sustained attention (Flehmig et al., 2007), which makes it an interesting measure with respect to the analysis of protocol conditions.

#### 2.3.1. Analytic methods

For all analyses, data were subset to stimulus conditions 1–4 (full stimulus sets), because conditions 5–8 (reduced stimulus sets) cannot be compared with 1–4 except in terms of physiological responses.

Main DVs included: the per block mean RT and RTV, each calculated from only correct response trials; *d*, a heuristic measure of accuracy per block (defined below); and the self-reported measure of difficulty (SRD) associated with stimuli, ranging from 1 = hardest to 6 = easiest. Independent variables (IVs) were factors describing the various stimulus and rule conditions.

Response times tend to follow Weibull or Gamma distributions (Palmer et al., 2011), rather than Gaussian. Thus, for modeling the effect of condition on response time performance, we used General Estimating Equations (GEE) which allow the specification of distributions alternative to normal (Højsgaard et al., 2006). GEEs allow relaxation of many of the assumptions of traditional regression methods such as normality and homoscedasticity, and provide the unbiased estimation of population-averaged regression coefficients despite possible misspecification of the correlation structure. GEEs estimate the *population behavior*, rather than model the conditional individual behavior; the estimates (βs) are expressed as log odds.

All GEE models used specified DV as RT or RTV; subject ID was used to identify clusters; the “family” was given as Gamma, link as “identity”; and the correlation structure was defined as “exchangeable.” WishGLD should induce a considerable learning effect, as participants practice across the various rule and stimulus conditions—all models thus control for time in the test, indexed by *block* number.

To derive a measure of accuracy, we applied formula 1 to obtain a heuristic “discrimination score” *d* for each block:

(1) $$\begin{array}{l} d=\frac{C+min\left(S,sss\right)}{T} \end{array}$$

where *C* is the number of correct responses per block, *S* is the number of “search” errors (occurring before the first correct response), *sss* is the search space size, and *T* is the total number of trials in the block (i.e., *C*+*S*+errors after first correct response).

For testing the hypotheses against accuracy, we took a different approach. We treated *d* as a classifier of the condition of interest per hypothesis, and tested the quality of classification using Receiver Operating Characteristic (ROC) curve methods. ROC curves facilitate visual examination of a classifier. If *d* acts as a significantly better classifier of a given condition than its counterpart, e.g., global faces vs. letters, it can be inferred that the condition difference has a systemic effect on participants' ability to discriminate the conditional stimuli. If there is no effect, then the ROC curve will be close to the diagonal line given by random choice, which can be visually checked and tested. For testing, the Area Under Curve (AUC) metric was calculated for all curves. AUC is related to the value of the Wilcoxon-Mann-Whitney *U* statistic by Equation (2), (Mann and Whitney, 1947). The resultant *z* score then allows significance testing and effect size (ES) calculation Field (2005).

(2) $$\begin{array}{l} z=\frac{AUC.n_{1}.n_{2}-\frac{n_{1}.n_{2}}{2}}{\sqrt{\frac{n_{1}.n_{2}\left(n_{1}+n_{2}+1\right)}{12}}} \end{array}$$

where *n*_1_, *n*_2_ are the sizes of the condition samples. It is important to be clear that these are not ROC curves of a classifier selecting between classes (e.g., faces and letters) in a single condition. Instead, they treat *d* as a classifier by *post-hoc* labeling of blocks from **separate** conditions. Thus, while the interpretation is clear, it is **not** *the same interpretation usually given to ROC curves*. For example, these curves cannot be inverted as per normal ROC curves. We thus manually assigned the true/false labels to produce curves with AUC >0.5, for consistent interpretation.

The value of all block-level metrics RT, RTV, and *d* are a function of multiple participant actions, which implies that extreme values are not likely to come from unintended activity or data entry errors. We therefore assume these behavioral block-level results have low noise, and remove outliers only for visualizations, not testing.

#### 2.3.2. Hypotheses

For testing the hypotheses H1a-c, H2a-b, we defined models as follows (non-specified GEEs are as above):

- H1a - to test the relationship of search and rule-found performance, we defined a GEE model for the DV of RT over “rule found” trials per block; IVs were: count of errors and RT over search trials, and block. Data was restricted to blocks where more than one search error had been made. We also visually examined the interactions between errors during search trials and mean RT from rule-found trials (**Figure 7**, top).
- H1b - to test the global precedence effect against the relationship of search and rule-found performance, we visually examined the interactions between errors during search trials and mean RT from rule-found trials, separately per stimulus level (**Figure 7**, bottom). We then tested the difference between the two vectors of mean RTs, fitting a linear model against the error sequence number.
- H1c - we tested performance metrics by comparing blocks with unchanged rule level from the previous block, against blocks with changed rule level. We modeled RT and RTV by GEE, with IVs for level switch (true/false), and block. We modeled *d* using ROC curves, with class labels for level switch (true/false); we tested for significance as described above.
- H2 - We explored the inter-individual variability in performance between stimulus classes by visually examining the relationship between the categorical SRD variable and the continuous performance data *d* and RT, and by testing the effect of subjectively experienced difficulty with a GEE model. The model was defined with RT as the DV, and target stimulus class and SRD value as the IVs, and the data was clustered per subject. To test, we compared a null model without SRD, and a full model with SRD as an explanatory variable.

#### 2.3.3. Protocol validation analysis

All testing of validation propositions followed similar procedures as described above, with the following specific model details:

For the GEE model testing Pr1a, IVs were factors for global feature, local feature, global × local interaction, and block. This model used the subset of data for stimulus conditions 1–4 and rule G1.obj. A similar GEE model was used for Pr1b, with the subset instead specifying rule L1.obj; and again for Pr2a,c but with the subset for rule G2.col; and for Pr2b,d but with the subset for rule L2.ori.

For testing Pr3a, the GEE model specified IVs of focus (global, local), and block; the subset of data was all rules. To test Pr3b, the GEE model specified IVs of rule (G1.obj, G2.col), and block; the subset of data was global focus blocks. For Pr3c, the GEE model IVs were rule (L1.obj, L2.ori), and block; the subset of data was local focus blocks. Data for all of Pr3a-c included stimulus conditions 1–4.

For Pr4 we examined all correct trials where the target-matching card was presented in the right or left position among the four reference cards (see Figure 4). We modeled correct trial RTs by GEE, with IVs for card position and the interaction of card position with global and local features and rule.

For Pr5, to tackle the question of learning, we used visual examination, plotting the group mean RT split by attention level for sets, blocks and trials.

Based on the number of tests run to validate the protocol, totalling 22, we performed Bonferroni-Holm familywise error rate correction, such that results marked significant in Table 3 below are with respect to the corrected test.

**Table 3:** **Results of statistical testing with RT and RTV as DV (dependent variable) for each validation proposition**.

**Prop**.	**Prediction**	**Confirmed?**	**DV**	**IV (binary)**	**Data for rule**	**β**	***SE***	**Waldχ^2^**	**adj. *p***
Pr1a	No effect	✓	RT	Global face/letter	G1.obj	0.08	0.05	1.4	1
Pr1b	No effect	✓	RT	Local shape/letter	L1.obj	0.04	0.05	0.8	1
Pr2a	No effect	✓	RT	Global face/letter	G2.col	−0.09	0.1	0.5	1
Pr2b	No effect	✗ ^***^	RT	Local shape/letter	L2.ori	−0.15	0.04	16.4	<0.001
Pr2c	No effect	✓	RT	Local shape/letter	G2.col	0.1	0.12	0.75	1
Pr2d	No effect	✓	RT	Global face/letter	L2.ori	−0.004	0.04	0.01	1
Pr3a	Difference	✓ ^***^	RT	Focus global/local	All	−0.37	0.02	392	<0.001
Pr3b	Difference	✓ ^***^	RT	Rule G1.obj/G2.col	G1.obj, G2.col	0.3	0.03	86	<0.001
Pr3c	Difference	✓ ^***^	RT	Rule L1.obj/L2.ori	L1.obj, L2.ori	−0.2	0.01	383	<0.001
Pr1a	No effect	✓	RTV	Global face/letter	G1.obj	0.5	0.5	0.9	1
Pr1b	No effect	✓	RTV	Local shape/letter	L1.obj	0.8	0.7	1.3	1
Pr2a	No effect	✓	RTV	Global face/letter	G2.col	2.3	1.3	3.2	0.9
Pr2b	No effect	✓	RTV	Local shape/letter	L2.ori	−0.12	0.6	0.04	1
Pr2c	No effect	✗ ^✝^	RTV	Local shape/letter	G2.col	2.7	1.0	7.4	0.08
Pr2d	No effect	✓	RTV	Global face/letter	L2.ori	0.3	0.7	0.2	1
Pr3c	Difference	✓ ^***^	RTV	Rule L1.obj/L2.ori	L1.obj, L2.ori	−1.6	0.3	29.3	<0.001

Significance level,

*** *p < 0.001*,

✝ *0.1 (marginal)*.

## 3. Results

**Figure 6:**
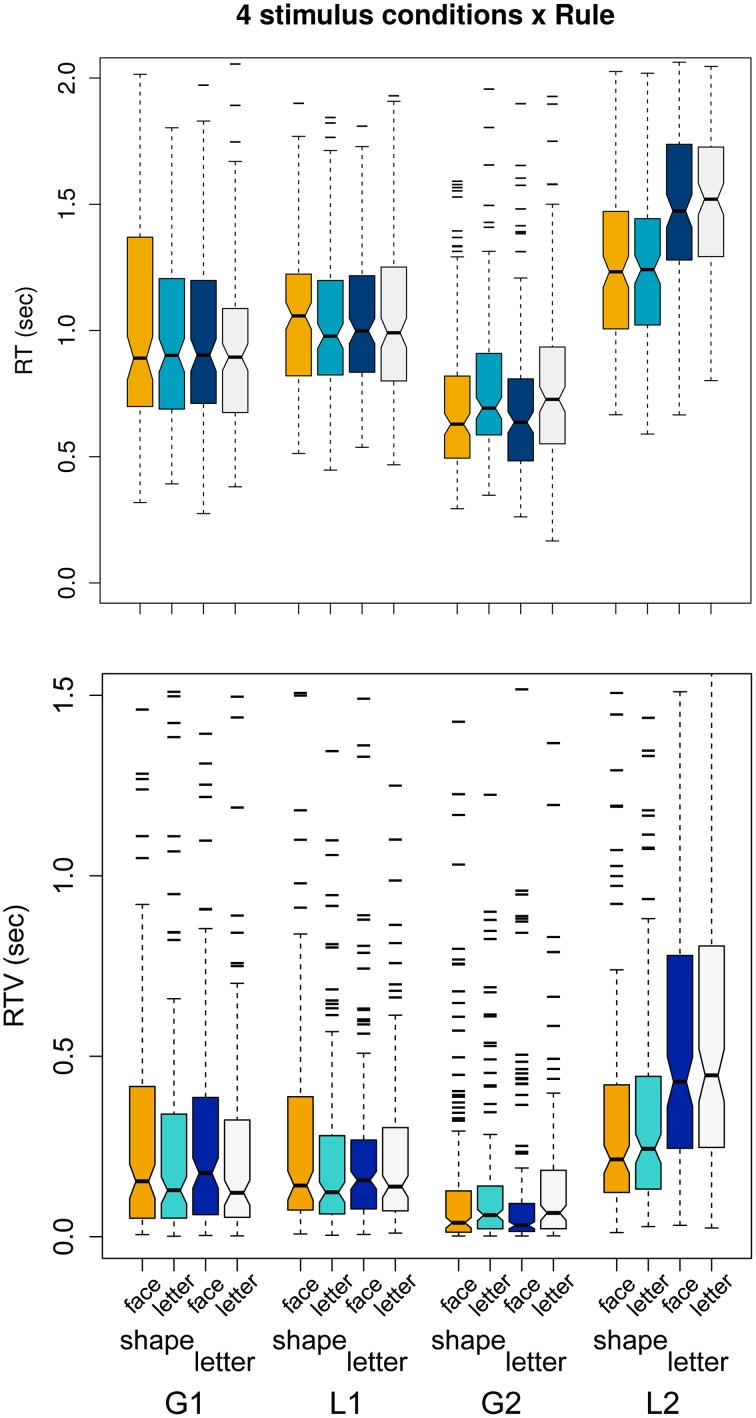
**Top panel:** mean RTs per condition. Rules G1.obj, L1.obj, G2.col, L2.ori are grouped in blocks of four, and colors within blocks correspond to stimulus conditions. **Lower panel:** as top panel, for RTV.

For a general perspective on the relationship between conditions and timing of responses, Figure 6 visually illustrates the group performance by speed and variability of responses, bearing on both the hypotheses (Section 3.1) and validation questions (Section 3.2). RT × condition is plotted in the top panel and RTV × condition in the lower panel.

The figure illustrates several results of interest. It is apparent that mean RTs are more alike within each rule than between rules. Ranking all conditions low-to-high by mean RT, we have G2(face) <G2(letter) <G1.obj <L1.obj <L2(shape) <L2(letter). This supports the *global precedence effect*, though in a novel context.

### 3.1. Experimental results

**Figure 7:**
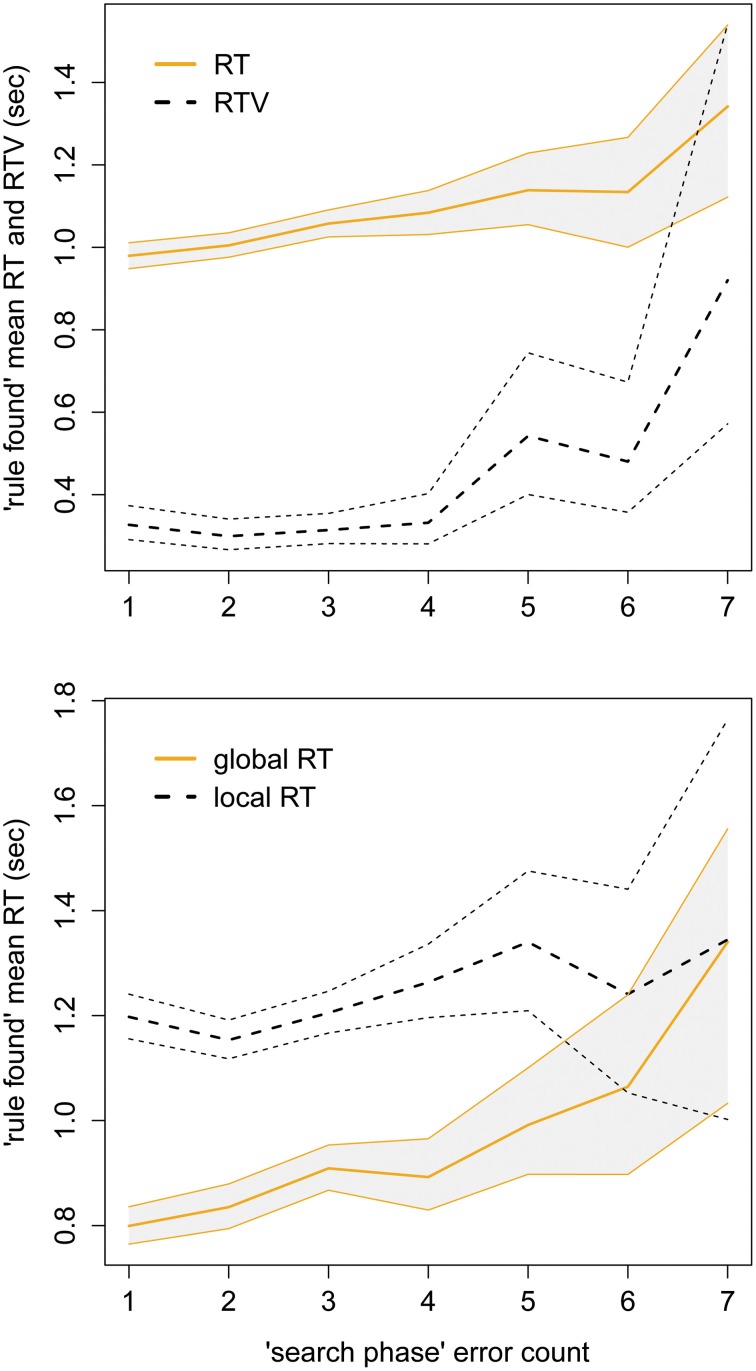
**Top panel:** mean RT, and RTV, of correct trials when the rule is found, plotted against errors during search, with bootstrapped 95% CI. **Lower panel:** mean RTs, abscissa and ordinate as top panel, split by global or local search focus.

The relationship between search-phase performance and rule-found performance, H1a, showed significant support by GEE model: search RT predicted rule-found RT, *p* < 0.001, β = −0.07, *SE* = 0.01, Waldχ^2^ = 45; as did search errors, *p* < 0.001, β = −0.02, *SE* = 0.002, Waldχ^2^ = 85. The effect of search errors on rule-found RT performance is illustrated in Figure 7, top panel, showing a clear increase in RT with more search errors.

For the question of global precedence, H1b, the difference between mean RTs per stimulus level trended toward zero as errors increased from one to seven. The trend was significant by *t*-test, *p* < 0.05, *t* = −2.9. The magnitude of change was −50 ms per error level. The convergence is also shown clearly by the plot in Figure 7, bottom panel, where the bootstrapped 95% confidence intervals overlap at the sixth error.

The question of switch cost, H1c, resulted in no visible differences or significant test results for RT or RTV. ROC curve testing of *d* gave AUC = 0.5 (indicating a curve very close to random chance); *p* < 0.001 was significant but at very low ES = 0.07.

**Figure 8:**
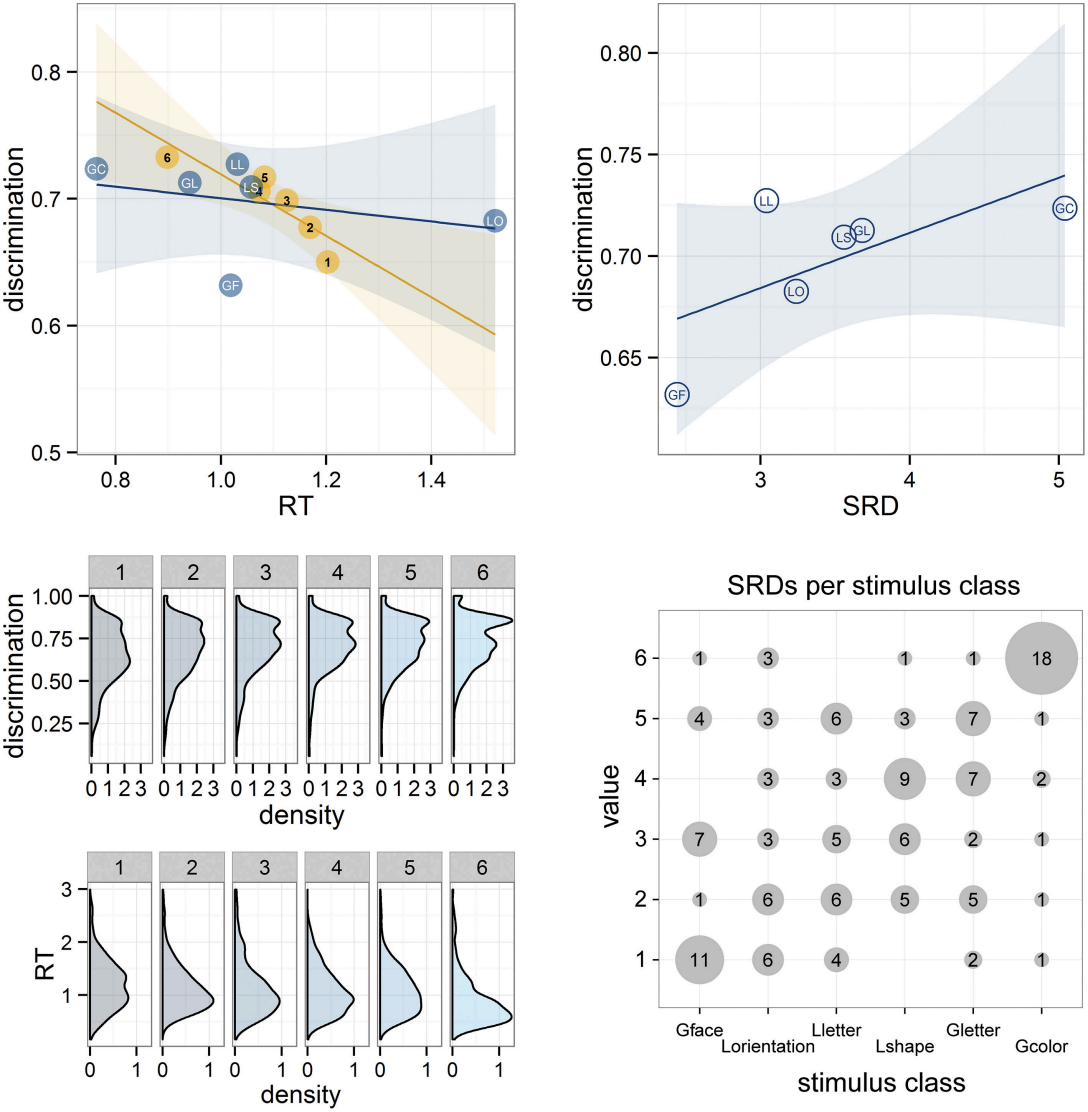
**Top left:** SRD (self-reported measure of difficulty) classes 1–6 (where 1 = hardest and 6 = easiest) and also target stimulus classes are scatter plotted in the space of *d*×RT. Target stimulus labels are GF, global faces; GL, global letters; GC, global color; LS, local shapes; LL, local letters; LO, local orientation. **Top right:** target stimulus classes scatter plotted in the space of *d*×mean SRD. Each group of points is fitted with a linear regression line with 95% CI band. **Bottom left:** density plot for six SRD classes of *d* (above) and RT (below). **Bottom right:** SRDs assigned to each stimulus class, with classes ranked left to right by average SRD value.

Regarding H2, we investigated the effect of subjectively experienced difficulty on performance by comparing null and full GEE models. The model estimates β = 0.013, *SE* = 0.004, and the analysis of the Wald statistic shows a significant effect (Waldχ^2^ = 9.83, *p* < 0.01). Figure 8 presents a scatterplot of subjective difficulty ratings' (SRD's) and stimulus classes' relationship to the two performance metrics: the response times and the discrimination metric. The figure shows that SRD levels have a good fit to objective performance, while the fit is poorer for target stimulus classes.

**Figure 9:**
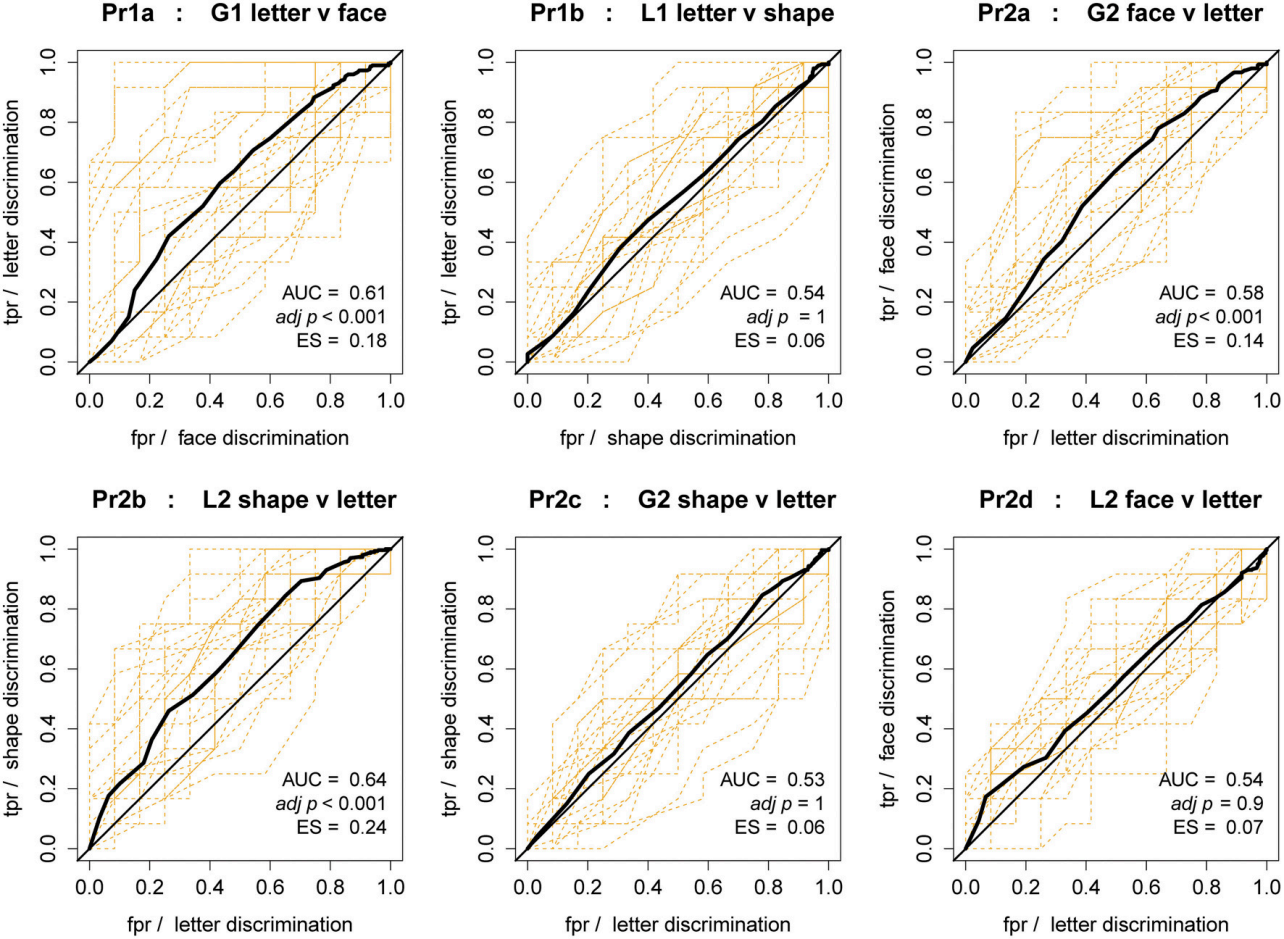
**ROC curves for discrimination score classification of stimulus condition**. Each figure corresponds to a hypothesis, and shows average curve as a thick black line, random chance level as a diagonal line, and curves per participant as dashed lines. Legend shows AUC (area under curve), significance of AUC (Bonferroni-Holm adjusted), and ES (effect size) of AUC in bottom right.

Additionally, per participant curves (dashed lines) in Figure 9 illustrates the inter-individual variability with respect to accuracy of matching-task performance (based on tests of the validation propositions, see below). This figure indicates that discrimination of visual features can be very sensitive to individual differences. While group average curves for stimuli pairings (each separate panel) are not very far from random (at the diagonal), many individual curves are quite far from random. In this context, these extreme curves indicate that the task itself acts as a good classifier of individual discriminatory accuracy.

### 3.2. Validation results

Results of statistical testing on RTs for each hypothesis, and on RTVs for each applicable hypothesis, are listed in Table 3. Model *p*-values have been corrected by Bonferroni-Holm and listed in adjusted form.

Results for Pr1a,b show clearly that *target* stimulus conditions do not affect RT or RTV in G1.obj, L1.obj tasks.

Regarding Pr2a,b, same-level non-target stimuli do not affect RT or RTV for rule G2.col, when the target is color. However, for rule L2.ori, orientation, the difference is significant at *p* < 0.0001, with the direction of relationship indicating RT(shape) < RT(letter). There is no L2.ori difference for RTV. And for Pr2c,d, other-level non-target stimuli do not affect RT for either of the tested rules. RTV is unaffected for L2.ori but not for G2.col, where the local shapes have induced greater RTV, though this is non-significant after correction.

For the question of between-rule effects, Pr3a-c, all hypotheses were supported by significant differences, *p* < 0.0001; where global rules had shorter RTs than local, color targets had shorter RTs than global faces/letters, and orientation targets had longer RTs than local shapes/letters. Pr3a,b are not defined for RTV. Pr3c was supported by a significant difference, *p* < 0.001, where orientation targets had larger RTV than local shapes/letters.

For the question of lateral presentations, Pr4, visual inspection of mean RT for all combinations of rule × stimulus conditions, comparing left- vs. right-hand presentations, found no comparative differences. No significant effects of card position were found in statistical tests, alone or by interaction.

**Figure 10:**
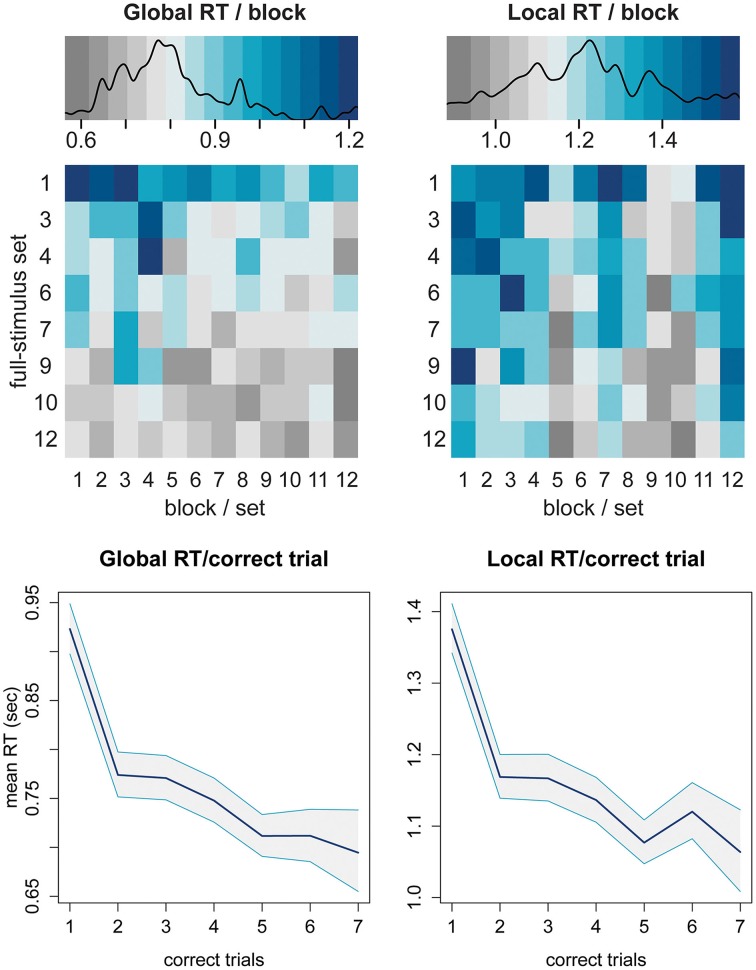
**Top panel:** mean RT per block × set, split by global and local. **Lower panel:** mean RT of each correct trial split by global and local, averaged over all blocks × set, with 95% bootstrapped CIs.

For Pr5, the effect of practice on RT is illustrated in Figure 10. The top panel shows RTs averaged over block × set, where each square is mean RT of all participants for given block (columns) in a given set (rows). For global attention, RTs visibly diminish over sets and blocks *within sets*, although it is not a monotonic trend. The local attention result is much less clear, as blocks 7 and 12 buck the trend: this was due to the accumulated effect of having several conditions where rule L2.ori was the target. The lower panel shows RT over correct trials; the trend over trials for both global and local is very clear.

Testing the validation propositions against the accuracy heuristic *d*, the ROC curves are shown in Figure 9. Visual inspection is enough to tell that Pr1b,Pr2c,d are supported, as the curves lie mainly on the diagonal; the statistical testing is also non-significant and of small effect size. In contradiction of Pr1a,Pr2a,b, testing found significant differences between stimulus conditions, for global targets, global and local same-level non-targets; although with minor effect sizes. Pr3a-c are not defined for *d*.

AUC, *p* and ES values are shown on each figure for the group-level curve, however we must be cautious in interpretation of these values. The Mann-Whitney-Wilcoxon method of *p*-value calculation is sensitive to sample size, and for group analysis our samples were on the order of hundreds. On the other hand, with smaller sample sizes the per-participant significance testing is more easily interpretable, and the differences must be much larger before significance. In these results only Pr1a and Pr2a had any individual curves significantly different to random after multiple comparison correction (5 or 20% for Pr1a, 0.6 ≤ ES ≤ 0.8; 1 or 4% for Pr2a, *ES* = 0.6). This supports the visual assessment of individual variability for H2, above.

Based on group and individual AUC-testing, it is possible that for the *d* measure of accuracy, Pr1a is not supported and faces are more difficult to accurately discriminate than global letters, though not at any cost in time-based metrics.

## 4. Discussion

Our core research questions concerned the interplay between complex cognition and global-local attention. We showed that when it was harder to *find* the rule (in terms of errors), subsequently *applying* the rule during the matching task was also harder, H1a. The global precedence effect was also responsive to search difficulty. We found no evidence for a switch cost associated with the attention levels, although we did not design specifically for this test. Finally, we saw that inter-individual variation has a strong effect on performance and that individuals' *post-hoc* reflection on the difficulty of each stimuli correlates with their performance.

In terms of validation, the protocol works largely as intended. Some issues were discovered by the thorough analysis, as discussed below in Section 4.4.

### 4.1. Hypotheses

The separate, but linked, metrics of RT and RTV clearly show (see Figure 7) the dependency that speed of response has on smooth updating of the rule during search phase, as per H1a. It is impossible to known *why* each search error was made, for numbers of errors >SSS (usually three). However, a logical, discriminating participant can always deduce the next rule by trial and error in < SSS+1 errors. Thus, it is reasonable to assume some deficit (temporary or otherwise) in the complex cognition of hypothesis updating, which bleeds over into the matching task, reflected in slower and much more variable response times. This could be due to reduced efficiency of hypothesis unpacking (Tversky and Koehler, 1994), i.e., not making use of the information available from feedback to update probabilities.

Regarding H1b, Figure 7's lower panel illustrates an interesting dynamic for the global precedence effect in a complex cognitive task. Although global processing is faster when participants are sure of the task (low search error count), when the error count goes up the RT for the two levels begins to converge. Larger CIs at higher error counts are certainly due to smaller sample sizes (201 blocks reached > 4 search errors); however the mean trend is clear. The global processing advantage has previously been shown to diminish with information lag (May et al., 1995), and constantly switching tasks (Kéïta et al., 2014); but has not been tested in a manner similar to ours, and to our knowledge this is the first finding that the effect would be modulated by subjective uncertainty. Interestingly, while (Navon, 2003) postulates that “good form” of the global figure is required for the global precedence effect to take place, our color stimuli have no such “form,” or pattern at all.

For H1c, as justified above, a cautious interpretation should be applied to the ROC result for switch cost. With very small ES, and no complementary effects for RT or RTV, we believe the significant *p*-value should be taken as a spurious result. Thus, there was no evidence of a cost, although this is not evidence of no cost. It may be the case that the rule search phase forced participants to abandon a fixed focus on a given attentional level on *every* block, preventing any advantage to same-level switches.

H2 was supported by a number of analyses. Looking at the self-report data in Figure 8 top left panel, participants' subjective difficulty rating SRD matches very well to both of the objective metrics *d* and RT, indicating two things: (a) participants accurately identified the difficulty of classes after testing, and consequently (b) we can infer that the performance in each class is due to their difficulty and not some other factor (e.g., mind wandering). The same result is shown in the density plots, where there is clearly agreement between the rating and the distribution of *d* and RT; moreover, ranks 2–5 are more distinct from the first and last rank than they are from each other, suggesting a clustering around the center. Further, the results with SRD likely indicate that while in general, the global precedence effect and stimulus complexity predict performance times per stimulus classes, there is inter-individual variance in perception/processing per different stimulus types, not totally dictated by low-level stimulus features, and that people can recognize their preference and evaluate their own performance. On the other hand, in the top left panel, the values for target stimulus classes are much less related to *d*: except for faces which is a *d* outlier. Secondary stimuli color and orientation are well separated from the primary stimuli in the RT dimension. The top right panel shows the interaction of the stimulus classes and SRD; here it is clear that faces and color are well separated from the other classes by their mean SRD score. However, the top left panel shows that color lies in the same range of *d*, and that faces lie in the same range of RT, as the main group of other stimuli. This implies that the subjective separation of color and faces in SRD is based on different dimensions of performance: color is recognized as easier because it is faster, not more accurate; faces are recognized as harder because of their lower accuracy, not because they were slower.

### 4.2. Validation

As a novel protocol WishGLD has been shown to meet the criteria for validation.

From Figure 6, the results for our “non-structural” stimulus conditions indicate that the simpler task of global color recognition has the fastest RTs, and the ontologically more complex orientation of local stimuli has the slowest (more complex because object recognition is required before orientation can be evaluated). Matching the predicted outcome, this result validates the use of multiple concurrent targets on each hierarchical level.

The means of conditions within the two “core” rules, G1.obj and L1.obj, are all equal. In other words, stimulus condition did not affect RT performance when tasked with matching global faces vs. letters, nor when matching local shapes vs. letters. G2.col and L2.ori rules showed more internal variance, with systematic bias: G2.col faces have smaller RTs than G2.col letters (not significant); for L2.ori orientation, shapes are faster to match than letters (significant). The advantage of G2.col faces over letters might be due to the face stimuli having a “centre of mass” more focal to the center of the card, compared to the global letters (D,J,L,U), whose “centre of mass” is distributed toward the card edges. The difference in decoding orientation may be due either to the semantic loading of the letters, which creates the impulse to orient the letters on a horizontal line for recognition; or to the ontological structure of letter orientation, as letters must be recognized as such before participants can apply the suggested strategy of mentally imaging the letter axis to evaluate their orientation.

The significant GEE results for RT did not show large β, absolute value range is 0.15 to 0.37 log odds; this translates to outcomes ranging from 8 to 18% more probable than their alternatives. On the other hand, β for RTV in Pr3c was −1.6 indicating a more skewed probability ratio for RTV. Given that RTV is inversely related to sustained attention, and in the context of Pr3c, the result implies that sustained attention is much greater for L1.obj (recognition of local objects) than for L2.ori (orientation).

From Figure 9, ROC curve analysis of the discriminability of stimulus conditions indicated three significant group results; however, we have suggested caution in interpretation of such *p*-values. Here, ES is a more trustworthy indicator than *p*-value, and ES never exceeded the 0.30 threshold of medium effect. Thus, it is probable that accuracy is relatively stable in the face of condition changes across the protocol, at the group level.

### 4.3. Object file theory

In studies reported so far, the hierarchical global-local stimuli have only presented a single target feature per level, resulting in simple object-files, potentially enabling the storage of multiple object-files per stimulus. Our study is the first to add multiple features at each level, making the resulting object-files more feature-rich, and as such, more resource-intensive. Based on our results in Pr2c-d, showing no effect of other level stimuli in any performance metric, it would seem that participants only attended to a single hierarchical level of the stimuli at a time. This suggests that they can only construct a single complex object-file, and thus only attend to either local or global features when classifying the cards. This would be hard to explain with the original DFF theory. A more explicit test would require simultaneous attendance of other level stimuli, but this is a matter for future work.

### 4.4. Future work

There is always room for improvement: some issues with WishGLD remain to be fixed. The size of the effect of L2.ori on RT is of some concern. Although it was expected, it is preferable to mitigate this bias. One strategy, that would improve the balance in difficulty between orienting local letters and shapes, is to mark letters and shapes with an accent such as a dieresis. The strength of this effect also highlighted a problem with the fixed-random configuration files for sets. As shown in Figure 10 (top), blocks 7 and 12 of every set had longer RTs than their neighbors. This was due to the random configuration files which had assigned the rule L2.ori to this block more times than any other rule. As a random distribution, the assignment of rules is not expected to be uniform, however such an effect can be controlled by generating random configuration files afresh for each participant.

Similarly, compared to global letters, the global faces were both subjectively more difficult and harder to discriminate (by *d*). This may have related to the unfamiliarity of the faces vs. semantic loading of the global letters. Future work might then try to generate more distinguishable faces, in order to balance the test in relation to letters, by introducing the faces more comprehensively before testing. Participants could be shown the original portraits alongside their AddRas cards in a pre-test familiarization. Alternatively, more distinctive faces such as caricatures could be used.

With respect to the object file theory of hierarchical processing, WishGLD could be adapted to do explicit testing of the exclusivity of hierarchical levels, for example with manipulation of other level stimuli controlling a GO-NOGO task structure.

Feedback stimulus could also be manipulated. Aversive stimuli such as displeasing noises (e.g., taken from the International Affective Digital Sounds database), could be matched to incorrect responses, contrasting with pleasant stimuli for correct responses.

### 4.5. Brain imaging

In the pending report on EEG data, we address the following research questions. First, we expect to replicate prior results that show asymmetry of cortical hemispheric activation related to processing of relative low vs. high spatial frequencies, when stimuli are “structural.” We expect this effect to be modulated, but not nullified, by the relative semantic value of the stimuli. We intend to further explore the relationship when target stimuli are non-structural, i.e., color and orientation. Given the trial structure of sequential target and reference presentations, we expect to find an effect across the fronto-parietal network sites, that elaborates the relationship between bottom-up perceptual filtering and top-down target (feature) selection. For the latter analysis we will use event-related methods.

An interesting area for future development is integration with other lines of research into cortical asymmetry and hierarchical processing. In both cases, interaction of parietal and frontal asymmetry via the fronto-parietal network (FPN) means recent work to characterize the FPN (Ptak, 2012; Zanto and Gazzaley, 2013) is of significant interest. The parietal locus of hierarchy-responsive asymmetry, and its dependence on executive attention, reinforces this. Essentially, the complex nature of cognition at this level allows us to rule nothing out, and indicates that study of hierarchy-related asymmetry should begin to be related to the study of the fronto-cortical asymmetries arising from cognitive constructs of motivation, emotion and attention (Gable and Harmon-Jones, 2008).

Regarding EEG asymmetry, frontal asymmetry has been linked to motivation by Gable and Harmon-Jones (2008) and others. Adapting the WishGLD protocol to manipulate motivation was considered during design. One obvious approach is gamification of the feedback structure, such that incorrect trials would be penalized, and participants would be incentivized either by extrinsic rewards or by a scoring comparison system (high score table). Such motivation could be manipulated between sets, introducing an extra two conditions, which could then be offset by removing some of the existing conditions, e.g., testing only faces and shapes.

Emotions have also been reported to interact with cortical asymmetry (Harmon-Jones et al., 2010), and emotional faces have been shown to affect local-global processing (Srinivasan and Hanif, 2010). Due to the use of face stimuli in WishGLD, it would be a simple matter to introduce emotional manipulation with emotional face conditions, again replacing a stimulus condition to maintain tractable total test time.

### 4.6. Conclusion

We have presented the WishGLD protocol as a variant of the well-known WCST. WishGLD concurrently tests executive control and global-local processing of visual stimuli. On the level of executive control, successful performance in the task requires the user to alternate between two “states”: searching for a new sorting rule after each rule update, and applying the found rule until the next update. The cards are designed to have both global level features (requiring integration of the features over the whole card) and local level features (requiring focused attention on local features). The task induces periods of divided attention or active level-switching, and periods of focused attention on one of the hierarchical levels, local or global.

The task is designed to (a) study a novel variant of global-local processing with extended sequences of selective attention, (b) control some of the confounds in earlier studies of hierarchical attention, and (c) better emulate typical real-world situations where local-global processing differences play a role, such as manipulating a user interface, driving a car, or interacting with our surroundings—situations where we routinely need to switch between observing the details and integrating the big picture.

To the best of our knowledge, this is the first study where each hierarchical level integrates multiple target categories. It is also the first study to have extended subject-induced states of selective attention, as opposed to task-induced attention paradigms. Additionally, the nature of the card sorting task allows modulation of factors such as executive control, workload and motivation, and the flexible design of the cards enables the modulation of emotional as well as perceptual factors (as described in Section 4.4).

Global-local dissociation has mostly been investigated using stimuli following the Navon letters (Bedson and Turnbull, 2002), with some exceptions (Fink et al., 1999). Although these studies have provided a wealth of knowledge, their stimuli are often too simplified to easily extend the results to natural environments, and the single dichotomy between one local and one global feature limits their use for studying inter- and intra-level effects.

WishGLD is a novel, validated protocol with the intention of controlling confounds and improving ecological validity. Further, as a tool for basic research, WishGLD has already illustrated several behavioral results of interest. The protocol thus has strong potential for future investigations, and we also welcome and expect replication and extension via the publicly available sources.

## Author contributions

KL conceived and developed the design, implemented the protocol, gathered and analyzed the data, and contributed to the draft. BC developed the design, implemented the protocol, gathered and analyzed the data, and wrote the draft.

## Funding

This work was partly supported by the Revolution of Knowledge Work project, no. 40228/13 and no. 5159/31/2014, funded by Tekes the Finnish Funding Agency for Technology and Innovation.

### Conflict of interest statement

The authors declare that the research was conducted in the absence of any commercial or financial relationships that could be construed as a potential conflict of interest.

## Footnotes

^1^Direct comparison of non-structural stimulus classes (color, orientation) is not considered feasible or necessary.

## Supplemental material

All code developed for building stimulus cards and running the WishGLD protocol, and the cards used in the experiment, can be found at https://github.com/bwrc/WishGLD.
